# Single amino acid change in tomato brown rugose fruit virus breaks virus-specific resistance in new resistant tomato cultivar

**DOI:** 10.3389/fpls.2024.1382862

**Published:** 2024-05-07

**Authors:** Zafeiro Zisi, Lucas Ghijselings, Elise Vogel, Christine Vos, Jelle Matthijnssens

**Affiliations:** ^1^ KU Leuven, Department of Microbiology, Immunology and Transplantation, REGA Institute, Division of Clinical and Epidemiological Virology, Laboratory of Viral Metagenomics, Leuven, Belgium; ^2^ Scientia Terrae Research Institute VZW, St.-Katelijne-Waver, Belgium; ^3^ DCM NV, Grobbendonk, Belgium

**Keywords:** tomato brown rugose fruit virus, tomato, cultivar, resistance breaking, resistance

## Abstract

**Introduction:**

Tomato cultivation across the world is severely affected by emerging plant viruses. An effective method for protection of commercial crops against viral threats is the use of cultivars harboring resistance genes. Tomato brown rugose fruit virus (ToBRFV), a recently emerged tobamovirus, is able to overcome the dominant *Tm-2^2^* resistance that is present in the majority of commercial tomato cultivars. In an effort to alleviate the severe consequences of ToBRFV on tomato production, tomato breeding companies are developing new cultivars with varying levels of resistance against ToBRFV.

**Methods:**

In the present study, cultivars with a new resistant phenotype against ToBRFV were screened against a wild-type isolate of ToBRFV, and subsequently, their performance under commercial greenhouse conditions was monitored. Following the identification of ToBRFV symptoms in a commercial greenhouse—where both new resistant and susceptible cultivars were interplanted—these cultivars were more closely examined.

**Results:**

The presence of ToBRFV was molecularly confirmed on both cultivar types suggesting that the new resistance had been broken. High-throughput sequencing (HTS) was used to study the complete genomes of viral isolates present in the two cultivar types. The analysis revealed a single amino acid change at position 82 of the movement protein of ToBRFV in the isolate present in the new resistant cultivar compared with the isolate identified in the susceptible cultivar.

**Discussion:**

A screening bioassay, that was performed to compare the infectivity of the two ToBRFV isolates, confirmed that only the isolate with this specific amino acid change could successfully infect the resistant cultivar, overcoming the new resistance against ToBRFV.

## 1 Introduction

Commercial tomato (*Solanum lycopersicum*) cultivation covers more than 5 million hectares worldwide, and its production in 2021 reached over 189 million tons, making it one of the most important vegetable crops for human consumption [FAOSTAT (Food and Agriculture Organization of the United Nations), 2023]. The crop can be heavily impacted by various plant viruses with several members of the genus *Tobamovirus* posing a major threat (Hanssen et al., 2010). The *Tobamovirus* genus includes viruses, such as tobacco mosaic virus (TMV) and tomato mosaic virus (ToMV), that can infect a wide range of plant families and cause serious damage to the crops (Spiegelman and Dinesh-Kumar, 2023). Its members have positive-strand RNA genomes of approximately 6.4 kb and rod-shaped virions (Ishibashi and Ishikawa, 2016). The tobamovirus genome has four open reading frames (ORFs): two replication proteins (RdRp), one of 126 kDa and its read-through derivative of 183 kDa, a 30-kDa movement protein (MP) and a 17.5-kDa coat protein (CP) (Ishibashi and Ishikawa, 2016).

A recent addition to the *Tobamovirus* genus is tomato brown rugose fruit virus (ToBRFV). After its first identification in 2014 in Israel (Luria et al., 2017) and subsequent isolation in 2016 in Jordan (Salem et al., 2016), the virus has managed to spread widely in a short time period. Currently, ToBRFV is present in Europe, North America, and Asia, mainly in the Middle East region (Zhang et al., 2022; EPPO, 2023). As is the case for most tobamoviruses, the main transmission route of ToBRFV is mechanical, through infected plant sap (Zhang et al., 2022). Transmission through seeds occurs at a low rate (Levitzky et al., 2019; Panno et al., 2020; Salem et al., 2022). The symptomatology of ToBRFV varies depending on the infected cultivar and the environmental conditions (Zhang et al., 2022). Some of its more characteristic symptoms include plant growth reduction, mosaic discoloration, deformation, and blistering of the leaves, as well as marbling, discoloration, and brown rugose on the fruits (Mehle et al., 2023). The virus endangers the economic feasibility of tomato production, with outbreaks causing huge yield and quality losses (Spiegelman and Dinesh-Kumar, 2023). Yield losses of 15%–55% have been reported (Avni et al., 2021) but can be even higher in case of crop eradication.

To protect commercial crops, plant genes providing resistance against tobamoviruses (R genes) have been identified and introgressed in tomato cultivars. More specifically, resistance is achieved by the R genes *Tm-1*, *Tm-2*, and *Tm-2^2^* (Pelham, 1966; Hall, 1980; Meshi et al., 1989; Lanfermeijer et al., 2003; Ishibashi et al., 2007; Hak and Spiegelman, 2021). *Tm-1*, previously identified in the wild tomato species *Solanum habrochaites*, encodes a protein that binds the tobamovirus RdRp, thus inhibiting RNA replication (de Ronde et al., 2014). Both *Tm-2* and its allele *Tm-2^2^* have been identified in *Solanum peruvianum* and encode proteins that interact with the tobamovirus MP (de Ronde et al., 2014; Hak and Spiegelman, 2021). The *Tm-2^2^*-derived resistance is a widely used, very durable, and effective method to control tobamoviruses, which is based on hindering the viral cell-to-cell movement and RNA transport through plasmodesmata (Weber and Pfitzner, 1998; Hak and Spiegelman, 2021). However, the emergence of ToBRFV is challenging conventional tobamo-resistance strategies, and recent studies have shown that this virus is able to circumvent the dominant resistance offered by the *Tm-2^2^* gene (Luria et al., 2017). This makes ToBRFV one of the most important threats in tomato cultivation in recent years (Zhang et al., 2022).

Due to the overwhelming pressure ToBRFV has put on the tomato cultivation industry, the identification of new resistances against ToBRFV to be introgressed in commercial cultivars has become a very important task for the tomato-breeding companies very early after its appearance. Many screening studies of wild *Solanum* species have been launched for the identification of resistant and tolerant genotypes (Zinger et al., 2021; Jewehan et al., 2022; Kabas et al., 2022). Presently, most tomato-breeding companies have developed new cultivars with varying levels of resistance against ToBRFV (Ykema et al., 2021; Varda et al., 2022; Gilan et al., 2023; Wanten, 2023).

In this study, tomato cultivars (Enza Zaden) with genomic sequences that were described in the deposit accession number NCIMB 43279 (Ykema et al., 2020), with a new resistant phenotype against ToBRFV, were screened against the virus. The new resistant cultivars were also monitored in commercial greenhouses in various planting schemes to evaluate their performance under practical conditions. In most cases the new resistant cultivars were planted next to other ToBRFV-susceptible cultivars, in the same greenhouse unit. In some of the commercial greenhouses that applied this mixed planting scheme, several months after planting, it was reported that ToBRFV symptoms were observed either in the susceptible or in the new resistant cultivars, or in both. Upon observation of ToBRFV symptom development in one of such greenhouses, samples were collected, and molecular analyses confirmed the presence of ToBRFV in both new resistant and susceptible cultivars suggesting that the new resistance was broken. This indication was further investigated via high-throughput sequencing (HTS) and screening bioassays.

## 2 Materials and methods

### 2.1 Tomato cultivar screening for resistance against a ToBRFV wild-type isolate

The screening bioassay was designed to test the level of resistance of the tomato (*S. lycopersicum*) cv. E15A.42917 against ToBRFV. Cultivar E15A.42917, as found in deposit accession number NCIMB 43279, harbors a new resistance against ToBRFV (Ykema et al., 2020). The ToBRFV-susceptible tomato cv. Climbo was used as a positive control for infection. At the first true leaf stage (10 days old), the seedlings were transplanted in plastic pots of 13-cm diameter and ToBRFV was inoculated on the cotyledons using carborundum as an abrasive.

Isolate 33610411 (MN882011.1) was used as a ToBRFV wild-type isolate (ToBRFV-WT), originating from the Netherlands in 2021, and purchased from the Deutsche Sammlung von Mikroorganismen und Zellkulturen (DSMZ) GmbH Collection of the Leibniz Institute. Isolate 33610411 was propagated in tomato plants cv. Climbo according to the instructions provided by DSMZ. Following propagation, fresh material was used to create the inoculum by homogenizing 1 g of young leaf material in 3 ml of phosphate buffer (pH 7.4) by vigorously shaking by hand with metallic beads. The same phosphate buffer (pH 7.4) was used for the mock inoculations of the infection negative controls.

The bioassay was conducted in a growth chamber under climate-controlled conditions. The light period was set to 14 h, during which the temperature was 23°C, and the dark period was set to 10 h, with a temperature of 19°C. The relative humidity inside the growth chamber was 70%.

The screening was set up in four treatment conditions that each included five plants: ToBRFV-susceptible plants mock inoculated, ToBRFV-susceptible plants inoculated with ToBRFV-WT, new resistant plants mock inoculated, and new resistant plants inoculated with ToBRFV-WT. At 14, 21, and 28 days post inoculation (dpi), the second youngest leaf of each plant was sampled and tested with reverse transcription quantitative real-time polymerase chain reaction (RT-qPCR) for ToBRFV detection. Statistical significance analysis on the observed differences in ToBRFV detection was performed using multiple linear regression analysis in R. At 28 dpi, the plants were scored for symptoms.

### 2.2 ToBRFV symptom scoring scale

ToBRFV symptom scoring in this study was performed according to a scoring scale developed in-house in the framework of the EU Horizon 2020 project, Virtigation. The scale assesses the presence and severity of four types of ToBRFV symptoms on the leaves of tomato plants: mosaic discoloration, blistering, deformation, and surface reduction (**Supplementary Figure 1**). The plants are scored for each symptom with an increasing severity scale ranging from 0 to 3. Score 0 corresponds to absence of symptoms, score 1 to mild symptoms, score 2 to moderate symptoms, and score 3 to severe symptoms (**Supplementary Figure 2**, mosaic discoloration example). The sum of the four score types is made to come to the total score, which can thus amount to a maximum of 12 for the most severely affected plants.

### 2.3 ToBRFV molecular detection

One hundred milligrams of leaf sample was collected and homogenized in 600 μl of Buffer RLT Plus, with 1% β-mercaptoethanol (RNeasy Plus kit, Qiagen, USA) using Bead Ruptor 24 (Omni International, Inc.) at 5.5 m/s for two cycles of 20 s with a dwelling step of 30 s between cycles, and PowerBead Tubes with 1.4-mm ceramic beads (Qiagen, USA). RNA was purified using the RNeasy Plus kit (Qiagen, USA).

Detection of ToBRFV was performed with the ISF-ISHI-Veg RT-qPCR protocol (ISF-ISHI-Veg, 2020).

### 2.4 Commercial greenhouse sampling

At the time that the study was performed, the new resistant cultivars were already commercially available. In agreement with the grower, commercial greenhouse 78 was monitored for the development of symptoms in new resistant cultivars, that had already been planted by the grower, after ToBRFV infection had occurred naturally in susceptible cultivars planted at the same location. More specifically, in this greenhouse, one compartment was planted with the susceptible cv. Sunstream, the new resistant cv. E15C.42785, and the new resistant cv. E15C.42788. All three cultivars share the same genetic background. The two new resistant cultivars, as found in deposit accession number NCIMB 43279, harbor the same resistance gene against ToBRFV in their genomes (Ykema et al., 2020).

The new resistant cultivars were not spatially separated from the susceptible one, whereby one row of new resistant cv. E15C.42785 and one row of new resistant cv. E15C.42788 were interplanted between the susceptible cv. Sunstream (**Supplementary Figure 3**). All cultivars were planted in September, and 5 weeks after planting, ToBRFV symptoms were detected in the susceptible plants after natural infection.

Upon observation of the first ToBRFV symptoms, leaf samples were collected from symptomatic susceptible plants and the closely neighboring non-symptomatic plants of both new resistant cultivars (**Table 1**). The samples were analyzed with RT-qPCR for ToBRFV presence to confirm the start of a ToBRFV outbreak in greenhouse 78.

**Table 1:** ToBRFV detection in the susceptible and new resistant samples collected from commercial greenhouse 78.

	Cultivar	Sample type	Cq value	ToBRFV detection
**1**	Susceptible	Leaf	7.67	ToBRFV detected, high concentration
**2**	E15C.42785 (New resistant cultivar)	Leaf	9.97	ToBRFV detected, high concentration
**3**	E15C.42788 (New resistant cultivar)	Leaf	25.46	ToBRFV detected, low concentration

### 2.5 Sample preparation and HTS

Two samples from greenhouse 78 were selected for high-throughput genome sequencing analysis, more specifically a symptomatic leaf sample from susceptible cv. Sunstream and a non-symptomatic leaf sample from the new resistant cv. E15C.42785, which both had low Cq values for ToBRFV (**Table 1**).

These samples were processed with the Novel Enrichment Technique of Viromes (NetoVIR) protocol (Conceição-Neto et al., 2015) to purify viral-like particles (VLPs) before sequencing. One hundred milligrams of leaf material was collected from each sample and homogenized in a Precellys Evolution Touch homogenizer, at 5,000 rpm for two cycles of 10 s with a dwelling step of 5 s, using 2.8-mm ceramic beads. The samples were then enriched for VLPs by centrifuging for 3 min at 17,000 *g* and filtrating the supernatant through a 0.8-µm PES filter, followed by a nuclease treatment step, using Benzonase nuclease (Millipore) and Micrococcal nuclease (New England Biolabs), to digest free floating nucleic acids. Nucleic acids protected by viral capsids were then extracted with the QIAamp Viral RNA mini kit (Qiagen, USA). The isolated nucleic acids were reverse transcribed and randomly amplified using the Whole Transcriptome Amplification kit (Sigma Aldrich). The PCR products were purified, and sequencing libraries were prepared using the NexteraXT Library Preparation Kit (Illumina) with unique double barcodes. The libraries were subsequently cleaned up with 1.8 ratio of Agencourt AMPure XP beads (Beckman Coulter, Inc.). Sequencing was performed on a NovaSeq6000 platform (Illumina) for 300 cycles (2 × 150-bp paired ends), resulting in approximately 26 million reads per sample.

### 2.6 Bioinformatics analysis of HTS-obtained ToBRFV sequences

The obtained sequencing data were processed using an in-house developed bioinformatics pipeline (De Coninck, 2021). The reads were trimmed using Trimmomatic (Bolger et al., 2014) by removing the first 19 bases of each read along with the sequencing adapters. Then, reads mapping to the sequenced negative controls, called contaminome, were removed from each sample using Bowtie2 (Langmead and Salzberg, 2012). After contaminome removal, the reads were *de novo* assembled using metaSPAdes (Nurk et al., 2017). The assembly was performed on the full-read dataset as well as on 10% and 1% of reads in parallel. This process allowed to circumvent the possibility of viral genomes breaking into smaller pieces during the assembly due to very high coverage (as observed previously in our lab). The contigs produced by the three assemblies were clustered together to remove redundant contigs. Clustering was performed by a combination of BLAST+ (Camacho et al., 2009) and CheckV (Nayfach et al., 2021). Finally, the resulting contigs were classified by DIAMOND (Buchfink et al., 2015) and KronaTools (Ondov et al., 2011) using the lowest common ancestor approach.

The nearly complete consensus genome sequences of ToBRFV obtained from the two samples were annotated and submitted to NCBI GenBank using Geneious Prime 2023.2.1 (https://www.geneious.com). The nearly complete ToBRFV genome assembled from new resistant cv. E15C.42785 sample was named “ToBRFV Greenhouse 78 non-resistance-breaking” and was submitted to NCBI GenBank as isolate “ToBRFV_G78_RB” with accession number OR760199. The nearly complete ToBRFV genome from the susceptible cv. Sunstream sample was named “ToBRFV Greenhouse 78 non-resistance-breaking” and was submitted to NCBI GenBank as isolate “ToBRFV_G78_NRB” with accession number OR760198.

The two sequences were aligned with each other and also with the NCBI ToBRFV reference sequence (NC_028478.1) and the sequence of ToBRFV isolate “ToBRFV-Tom2M-Jo” (MZ438228.1), an isolate breaking resistance against ToBRFV that was identified in wild *Solanum* species (Jewehan et al., 2022). On the nucleotide level, a multiple sequence alignment was performed using MAFFT v7.453 (Katoh and Standley, 2013) followed by the identification of the nucleotide substitutions between the sequences using SNP sites (Page et al., 2016). For the comparison on the amino acid sequence level, MAFFT v7.453 was used for the alignment, and the —clustalout option (Katoh and Standley, 2013) was selected to obtain the results in the clustal format to study the identified amino acid changes. The results of the sequence comparison were visualized using an in-house R script.

The sequence comparison at genome positions with interesting nucleotide substitutions was subsequently extended to include all nearly complete ToBRFV genome sequences (n = 217) available on the NCBI GenBank database (visited on 30/06/23). The sequences were downloaded and aligned with the two newly assembled consensus ToBRFV sequences using MAFFT v7.453. The results were visualized using AliView (Larsson, 2014).

### 2.7 Infectivity evaluation of the newly identified isolates ToBRFV_G78_RB and ToBRFV_G78_NRB in a new resistant cultivar

The infectivity of the two isolates identified in the greenhouse 78 was assessed on the previously screened new resistant cv. E15A.42917, which harbors the same resistance against ToBRFV in its genome as new resistant cv. E15C.42785, as found in deposit accession number NCIMB 43279 (Ykema et al., 2020). The ToBRFV susceptible cv. Mattinaro was used as a positive control for infection because it shares the same genetic background with new resistant cv. E15A.42917.

The bioassays were conducted in a growth chamber under the controlled conditions described in Section 2.1.

The samples containing ToBRFV_G78_RB and ToBRFV_G78_NRB were also co-infected with the CH2 genotype of pepino mosaic virus (PepMV). Therefore, the two ToBRFV isolates were purified to remove PepMV before their use in the bioassay to ensure correct assessment of their effect on the plants. The method used for the purification of isolates ToBRFV_G78_RB and ToBRFV_G78_NRB was optimized in the framework of the EU Horizon 2020 project, Virtigation. More specifically, each initial sample was used to create an inoculum by homogenizing 1 g of leaf material in 3 ml of phosphate buffer (pH 7.4) with metallic beads. The homogenized material was used to mechanically inoculate tobacco plants (*Nicotiana tabacum xanthi*). The inoculations were performed using carborundum. ToBRFV infection on *N. tabacum xanthi* led to the formation of lesions on the leaves, which were then excised using a Stanley knife to isolate the localized ToBRFV. Each lesion was transferred to a separate PowerBead Tube with ceramic beads 1.4 mm (Qiagen, USA) and homogenized in 250 µl of phosphate buffer (pH 7.4) using Bead Ruptor 24 (Omni International, Inc.). The homogenates were used to inoculate ToBRFV-susceptible cv. Climbo tomato plants of 10 days old. The inoculations were performed on the cotyledons using carborundum. At 14 dpi, the susceptible plants were sampled, and the presence of ToBRFV and absence of PepMV were confirmed with RT-qPCR. Analysis of PepMV was done using RT-qPCR with specific primers for the CH2 genotype of the virus as described previously (Gutiérrez-Aguirre et al., 2009).

After confirming the purity of the ToBRFV isolates, RT-PCR and Sanger sequencing were used to confirm HTS results.

The purified material was used to create an inoculum as described in Section 2.1 by homogenizing 1 g of leaf material in 3 ml of phosphate buffer (pH 7.4) by vigorously shaking by hand with metallic beads. The same phosphate buffer was used for the mock inoculations of the negative infection controls. Inoculations were performed using carborundum as an abrasive.

This bioassay was conducted in two subsequent experiments, The first experiment was performed using isolate ToBRFV_G78_RB. It was set up in four treatment conditions that each included five plants as follows: ToBRFV-susceptible plants mock inoculated, ToBRFV-susceptible plants inoculated with ToBRFV_G78_RB, new resistant plants mock inoculated, and new resistant plants inoculated with ToBRFV_G78_RB. At 14, 21, and 28 dpi, the second youngest leaf of each plant was sampled and tested with RT-qPCR for ToBRFV detection. Statistical significance analysis on the observed differences in ToBRFV detection was performed using multiple linear regression analysis in R. At 28 dpi, the plants were scored for symptoms, and the presence of the nucleotide substitution at position 5156 on the ToBRFV_G78_RB-inoculated plants was confirmed with RT-PCR and Sanger sequencing.

The second experiment was performed using isolate ToBRFV_G78_NRB and was also set up in four treatment conditions as follows: ToBRFV-susceptible plants mock inoculated (treatment included three plants), ToBRFV-susceptible plants inoculated with ToBRFV_G78_NRB (treatment included five plants), new resistant plants mock inoculated (treatment included three plants), and new resistant plants inoculated with ToBRFV_G78_NRB (treatment included five plants). The sampling schedule, ToBRFV detection method and statistical analysis, and symptom scoring point were the same as the first experiment of the bioassay.

## 3 Results

### 3.1 New resistant tomato cultivar shows resistance against a ToBRFV wild-type isolate

The aim of the bioassay was to assess the level of resistance of the new resistant cv. E15A.42917 against a wild-type isolate of ToBRFV (ToBRFV-WT). To that end, susceptible and E15A.42917 plants were inoculated with ToBRFV-WT using mock inoculations as a control. The level of resistance was evaluated using RT-qPCR with ToBRFV-specific primers on young plant leaves and through observation of symptoms on the plants. Each inoculation experiment was performed once.

**Figure 1:**
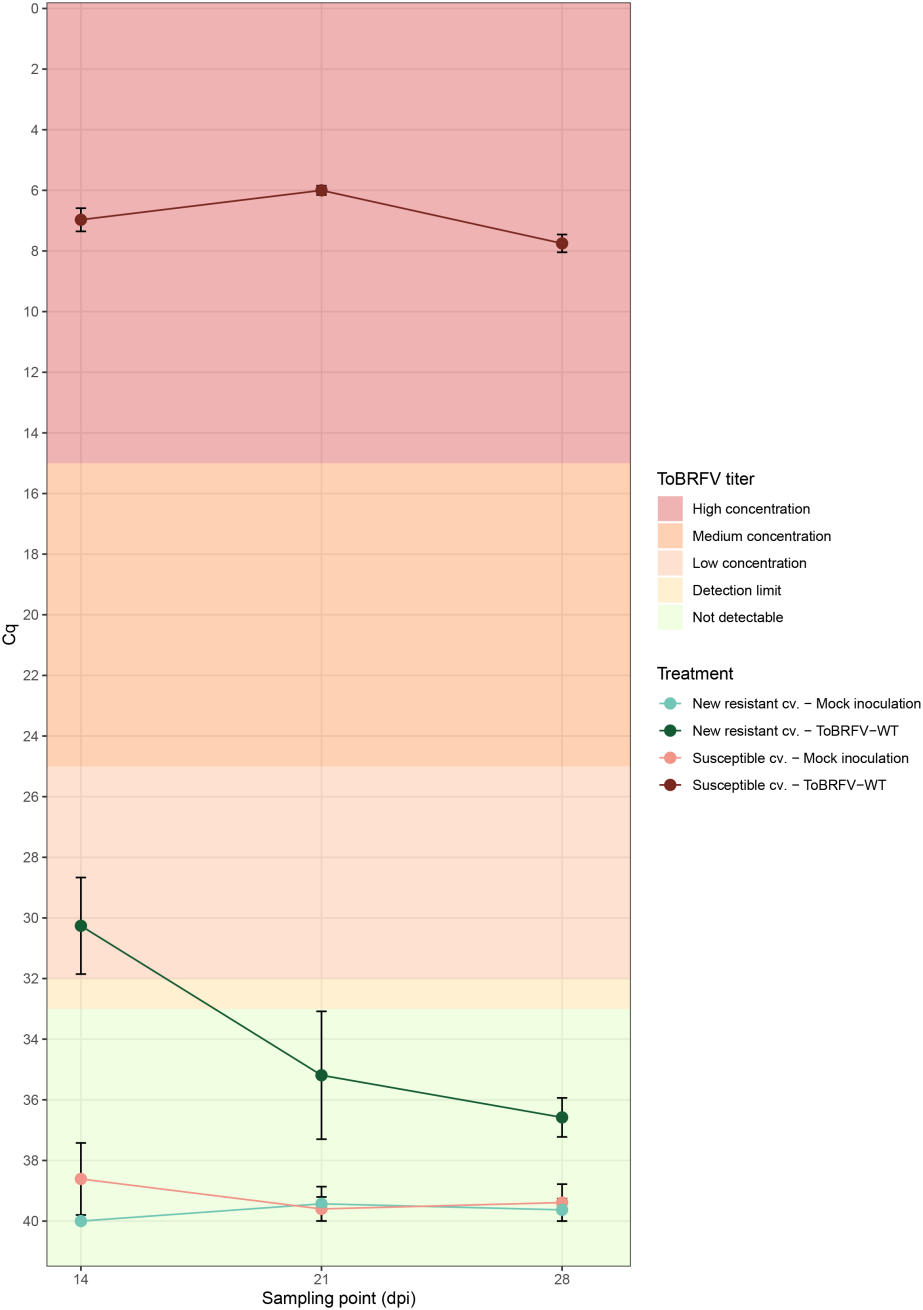
New resistant cultivar RT-qPCR screening against ToBRFV-WT—viral amplification. x-axis: Sampling point in days post inoculation. y-axis: Cq values from the ISF-ISHI-Veg RT-qPCR. Plotted values correspond to the mean of five Cq values measured at each sampling point for the five plants included in each treatment. The standard error for each measurement is indicated with error bars. Each treatment is plotted in a different color and shade combination: red color for the susceptible cultivar treatments and green color for the new resistant cultivar treatments; light shades for the mock inoculation treatments and dark shades for the ToBRFV-WT inoculation treatments. The plot’s background colors indicate the Cq range attributed to each ToBRFV titer tier.

The mock inoculation treatments of ToBRFV-susceptible (n = 5) and new resistant plants (n = 5) were used as healthy reference. The phenotype of the mock-inoculated new resistant plants served as a baseline for the evaluation of symptom development and scoring. ToBRFV was not detected using RT-qPCR in these treatments demonstrating the absence of contaminations (**Figure 1**). Symptom scoring was performed at 28 dpi. None of the control plants had developed any ToBRFV symptoms (**Supplementary Figure 4**, **Supplementary Table 1**).

**Figure 2:**
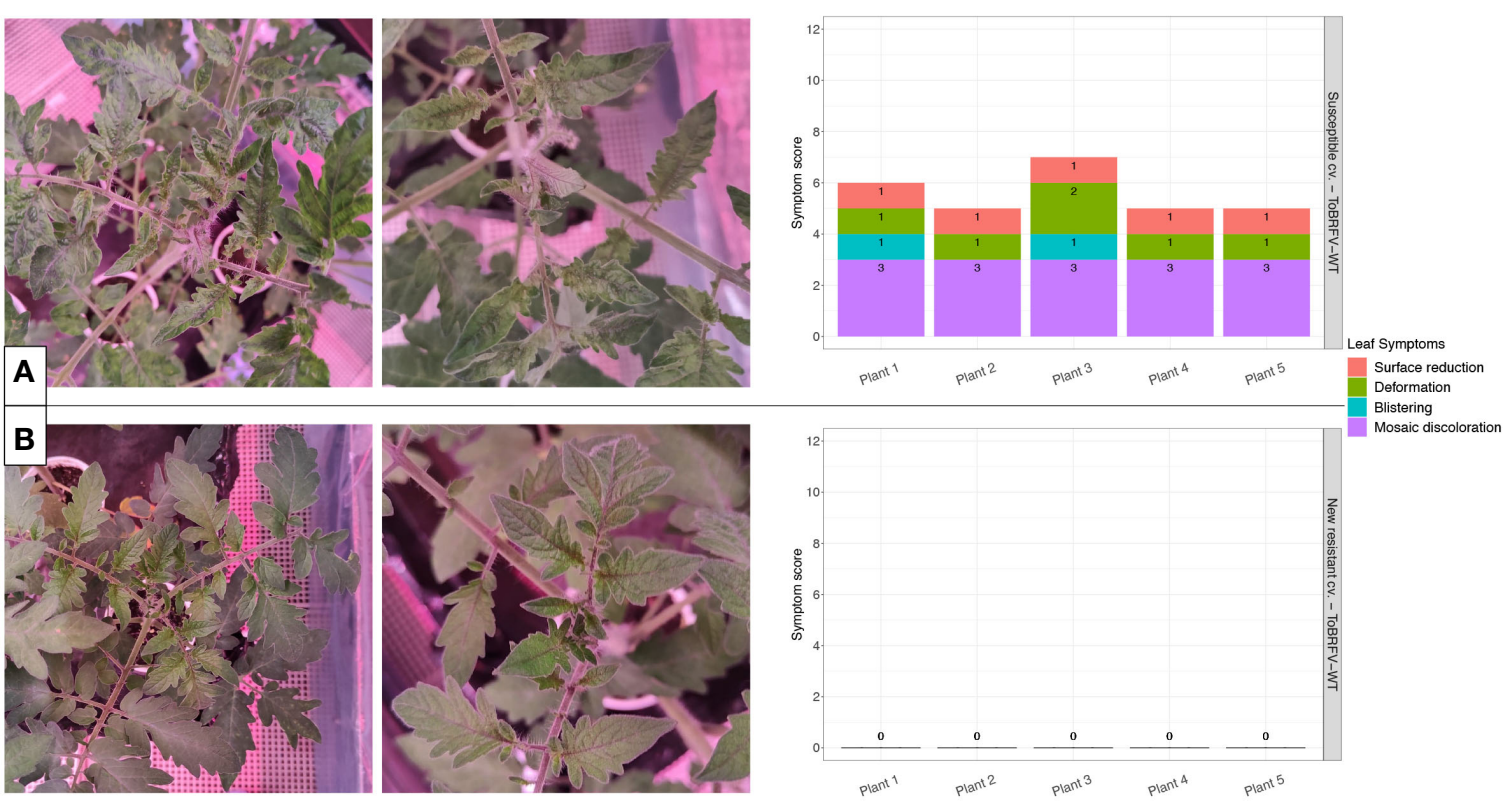
New resistant cultivar screening against ToBRFV-WT—leaf symptom development. **(A)** ToBRFV-susceptible plants inoculated with ToBRFV-WT, at 28 dpi. Left: representative pictures for leaf symptom development: Plant displays severe mosaic discoloration symptoms (light and dark green patches), as well as moderate deformation (narrow and elongated leaves), mild blistering, and mild surface reduction (in comparison with the infection-negative controls for the experiment). Right: symptom scoring per plant included in this treatment. **(B)** New resistant cv. E15A.42917 plants inoculated with ToBRFV-WT at 28 dpi. Left: representative pictures for leaf symptom development: No ToBRFV leaf symptoms were observed on the plant. Right: symptom scoring per plant included in this treatment. x-axis: plants included in each treatment. y-axis: overall symptom scoring. Each leaf symptom is plotted in a different color. The score for each symptom is included in black on each bar.

The ToBRFV-susceptible plants (n = 5) inoculated with ToBRFV-WT were used as a positive control for ToBRFV infection. Already at the first sampling point (14 dpi), low ToBRFV Cq values were detected in all the plants. These Cq values remained stable for this treatment at the following sampling points (**Figure 1**). All plants had developed leaf symptoms at 28 dpi. More specifically, all five plants displayed severe mosaic symptoms, and two plants showed mild blistering. Additionally, all plants displayed mild or moderate leaf deformation and a mild leaf surface reduction (**Figure 2A**, **Supplementary Table 1**).

The screened new resistant plants (n = 5) inoculated with ToBRFV-WT showed resistance against the virus under the tested conditions. At 14 dpi, relatively high Cq values, were detected on the HR plants (**Figure 1**). On the following sampling points, no ToBRFV presence could be detected (**Figure 1**). The Cq values detected in the new resistant plants inoculated with ToBRFV-WT were significantly different from the Cq values of the susceptible plants inoculated with ToBRFV-WT (p-value: 1.56e−08) throughout the bioassay. None of the plants developed any ToBRFV symptoms throughout the experiment (**Figure 2B**, **Supplementary Table 1**).

### 3.2 Detection of ToBRFV infections in new resistant and susceptible cultivars in a commercial greenhouse

The performance of new resistant cultivars, containing the same resistance against ToBRFV in their genome as new resistnat cv. E15A.42917 (Ykema et al., 2020), was monitored under practical conditions in greenhouse 78. Five weeks after planting, ToBRFV symptoms were observed in the susceptible cultivar that was interplanted with the new resistant cv. E15C.42785 and new resistant cv. E15C.42788 (**Supplementary Figure 3**).

Upon symptom development in this susceptible cultivar, leaf samples were collected from all three cultivars and tested with RT-qPCR to assess the spread of ToBRFV in the compartment. The symptomatic leaf sample collected from the susceptible cv. was found to have low Cq values for ToBRFV. The asymptomatic leaf samples from new resistant cv. E15C.42785 and new resistant cv. E15C.42788 showed low and high ToBRFV Cq values, respectively (**Table 1**). These results suggested that the ToBRFV infection occurred also in the new resistant cultivars planted in greenhouse 78.

### 3.3 Sequence analysis of ToBRFV isolates from infected new resistant and susceptible cultivars collected from a commercial greenhouse shows unique amino acid change associated with resistance breaking

One sample from the susceptible cultivar and one from new resistant cv. E15C.42785, which displayed low Cq values for ToBRFV based on RT-qPCR analyses (**Table 1**), were selected for genome analysis with Illumina HTS.

The HTS and subsequent bioinformatics analysis of the new resistant cv. E15C.42785 sample led to the assembly of a nearly complete ToBRFV genome (6,363nt). The sequence was named “ToBRFV Greenhouse 78 non-resistance-breaking” and it was submitted to NCBI GenBank as isolate “ToBRFV_G78_RB” (OR760199). The HTS and subsequent bioinformatics analysis of the susceptible sample also led to the assembly of a nearly complete ToBRFV genome (6,363nt). The sequence was named “ToBRFV Greenhouse 78 non-resistance-breaking,” and it was submitted to NCBI GenBank as isolate “ToBRFV_G78_NRB” (OR760198). Both sequences have four open reading frames encoding the viral small replicase subunit, RdRp, MP, and CP.

**Figure 3:**
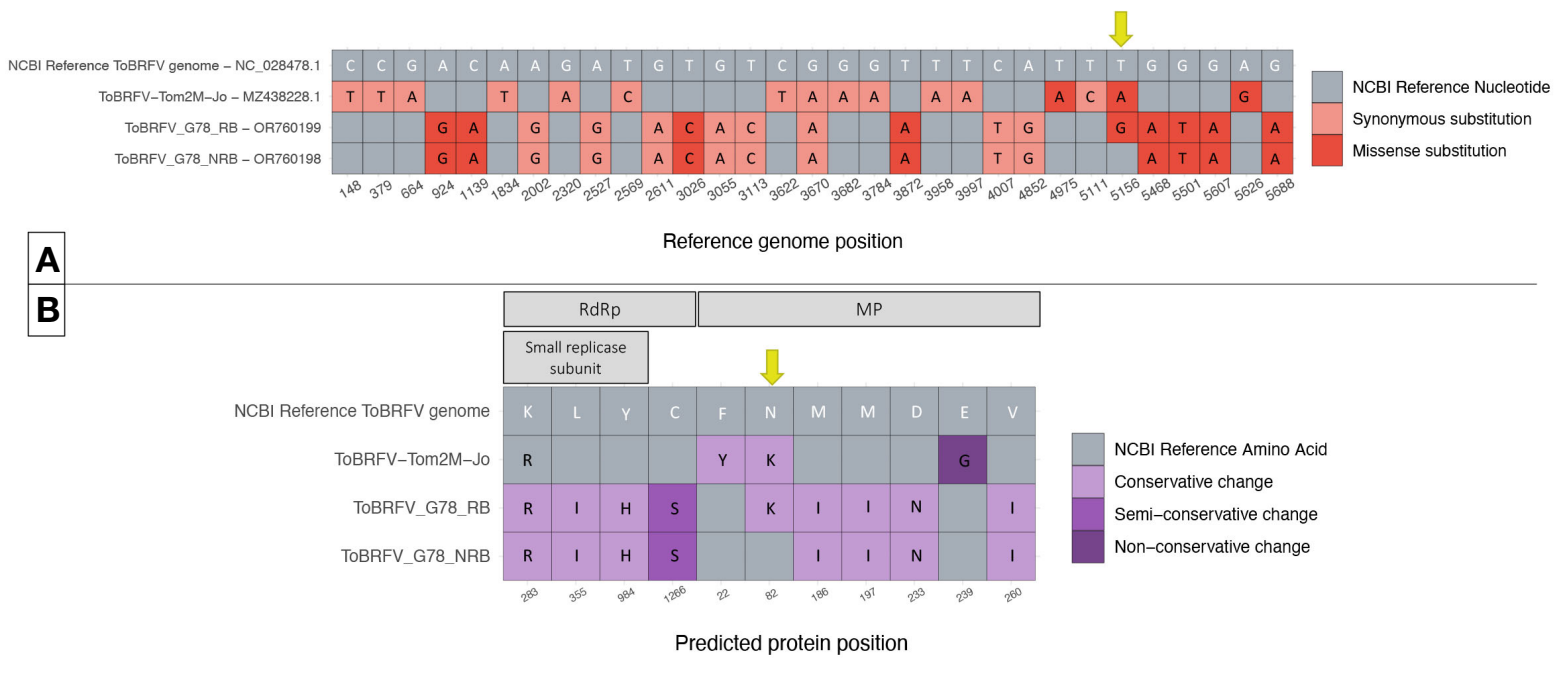
Comparison of ToBRFV_G78_RB, ToBRFV_G78_NRB and ToBRFV-Tom2M-Jo to the NCBI ToBRFV Reference sequence. **(A)** Nucleotide sequence comparison of coding parts of the ToBRFV genomes. The table displays only the genome positions where substitutions were identified in comparison with the NCBI ToBRFV reference sequence. Each nucleotide substitution is shown as a tile, and the corresponding reference genome position is indicated at the bottom of the table. The substitution type is plotted in color: Gray color for absence of substitution, pink color for synonymous substitutions, red color for missense substitutions. The reference nucleotides are included as white letters on the NCBI ToBRFV reference sequence tiles. The alternative nucleotides, when present, are included as black letters on the tiles of the isolate sequence they belong to. Position 5,156 with the substitutions of interest is indicated with a yellow arrow. **(B)** Comparison of the predicted protein amino acid sequences. The table displays only the protein positions where amino acid changes were identified in comparison with the NCBI ToBRFV reference protein sequences. Each amino acid change is shown as a tile, and the corresponding predicted protein is indicated at the top of the table, and the corresponding predicted protein position is indicated at the bottom of the table. The amino acid change type is plotted in color: gray color for absence of change, lilac color for conservative changes, purple color for semi-conservative changes, and deep purple color for non-conservative changes. The reference amino acids are included as white letters on the NCBI ToBRFV reference sequence tiles. The alternative amino acids, when present, are included as black letters on the tiles of the isolate predicted protein sequence they belong to. MP position 82 with the changes of interest is indicated in a yellow arrow.

The protein-coding parts of the two newly identified ToBRFV nucleotide sequences (genome positions 65–6,181 for both sequences) were compared to each other, as well as to the protein coding parts of the NCBI ToBRFV Reference nucleotide sequence (NC_028478.1, genome positions 77–6,193), and to the protein coding parts of nucleotide sequence of isolate ToBRFV-Tom2M-Jo, which was described in literature as a ToBRFV isolate breaking the ToBRFV resistance identified in wild *Solanum* species (MZ438228.1, genome positions 77–6,193) (Jewehan et al., 2022). A comparison of predicted amino acid sequences of the previously mentioned isolates was also performed. The NCBI ToBRFV Reference sequence was used as the comparison reference on nucleotide (**Figure 3A**) and amino acid level (**Figure 3B**).

The nucleotide level comparison of the two newly identified sequences showed one single missense substitution. The substitution was identified at genome position 5,144, which aligned with the NCBI reference genome position 5,156 in the MP coding region. In isolate ToBRFV_G78_RB, a T was substituted by a G (**Figure 3A**). The comparison of the predicted MP amino acid sequences of the two isolates showed an amino acid change from Asn to Lys located at position 82 of the MP. This was the only amino acid change found at the protein level comparison of the isolates, as was expected by the comparison of the nucleotide sequences (**Figure 3B**).

Each of the two newly identified isolates was also compared to the NCBI reference sequence for ToBRFV. The comparison on the nucleotide level showed 16 substitutions for ToBRFV_G78_NRB. For ToBRFV_G78_RB, the same 16 nucleotide substitutions were identified plus the additional T to G missense substitution at NCBI reference genome position 5,156 (**Figure 3A**). Comparison of the predicted protein sequences showed eight amino acid changes for ToBRFV_G78_NRB compared to the reference sequence, four located in the RdRp and five in the MP. ToBRFV_G78_RB had nine amino acid changes of which eight were identical to those of ToBRFV_G78_NRB. The additional amino acid change resulted in an Asn to Lys change at position 82 of the MP, which was uniquely identified on this isolate (**Figure 3B**).

Interestingly, the previous molecular characterization of isolate ToBRFV-Tom2M-Jo in literature (Jewehan et al., 2022) suggested that the breaking of resistance was the result of two missense nucleotide substitutions at NCBI reference genome positions 4,957 and 5,156. Those substitutions resulted in two amino acid changes in the MP of the isolate. More specifically at MP position 22, a Phe changed to Tyr, and at MP position 82, an Asn changed to Lys. The nucleotide and predicted amino acid sequences of this isolate were subsequently added to the comparison (**Figure 3**). On the nucleotide level, ToBRFV-Tom2M-Jo showed 16 substitutions compared to the NCBI reference sequence for ToBRFV and 30 compared to both ToBRFV_G78_RB and TOBRFV_G78_NRB (**Figure 3A**). The comparison on the predicted protein sequences showed 3 amino acid changes compared to the NCBI reference sequence for ToBRFV, 11 changes compared to ToBRFV_G78_NRB, and 19 changes compared to ToBRFV_G78_RB (**Figure 3B**). At NCBI reference genome position 5,156, isolate ToBRFV-Tom2M-Jo had a T to A missense substitution instead of the T to G found in the ToBRFV_G78_RB. Nevertheless, both substitutions resulted in the identical Asn to Lys amino acid change at position 82 of the predicted MP protein sequence, for both ToBRFV-Tom2M-Jo and ToBRFV_G78_RB.

Finally, all the publicly available ToBRFV full genome sequences (n = 217) in NCBI GenBank (data downloaded on 30/06/23) were aligned to the NCBI reference sequence showing that this missense substitution at reference position 5,156 was unique to isolates ToBRFV_G78_RB and ToBRFV-Tom2M-Jo (**Supplementary Figure 5**).

### 3.4 Infectivity evaluation of the newly identified isolates ToBRFV_G78_RB and ToBRFV_G78_NRB in a new resistant cultivar confirms resistance breaking phenotype of ToBRFV_G78_RB

The detection of isolate ToBRFV_G78_RB in new resistant plants in commercial greenhouse 78 suggested that a resistance breaking adaptation occurred. A bioassay was performed to verify that isolate ToBRFV_G78_RB indeed had overcome the new ToBRFV resistance as found in deposit accession number NCIMB 43279 (Ykema et al., 2020). The bioassay, performed with the new resistant cv. E15A.42917, was executed in two subsequent experiments. The first experiment was performed using the ToBRFV_G78_RB isolate to inoculate susceptible and new resistant plants using mock inoculations as a control. For the second experiment, the same set up was followed using isolate ToBRFV_G78_NRB. The two isolates originated from the same greenhouse and had identical nucleotide sequences with the exception of one missense nucleotide substitution that resulted in an amino acid change in the MP protein. Thus, the subsequent experiment allowed the assessment of whether the observed resistance breaking could be attributed to the single amino acid change. The infectivity and pathogenicity of the isolates were evaluated by RT-qPCR with ToBRFV-specific primers on young plant leaves and scoring of the ToBRFV symptoms on the plants.

**Figure 4:**
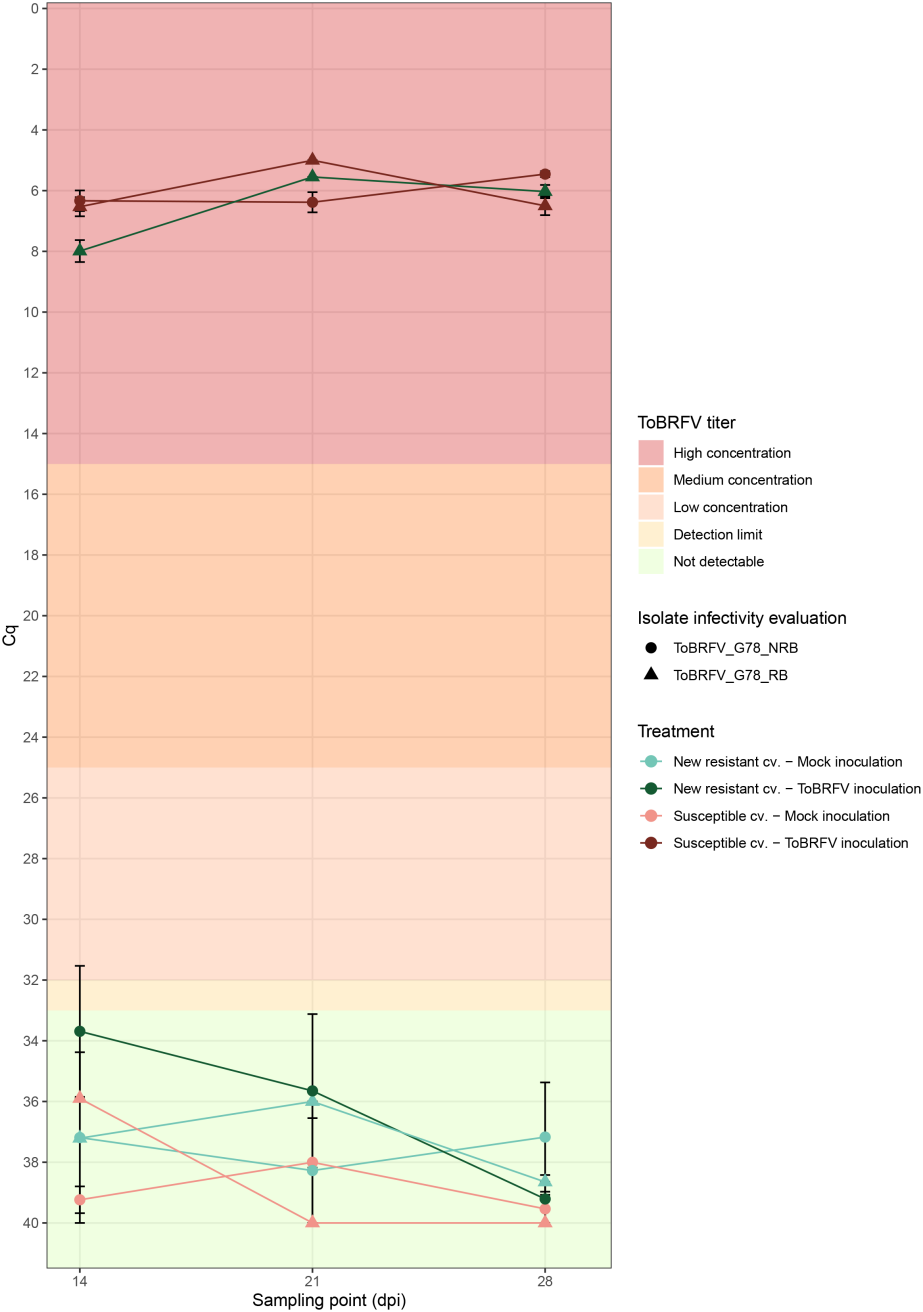
Infectivity evaluation of the newly identified isolates ToBRFV_G78_RB and ToBRFV_G78_NRB in a new resistant cultivar—viral amplification. x-axis: sampling point in days post inoculation. y-axis: Cq values from the ISF-ISHI-Veg RT-qPCR. For the infectivity evaluation of isolate ToBRFV_G78_RB (first experiment): plotted values for mock inoculation treatments correspond to the Cq value measured for the single mixed leaf sample collected at each sampling point for the five plants included in each treatment, plotted values for viral inoculation treatments correspond to the mean of five Cq values measured at 14 and 28 dpi for the five plants included in each treatment, and to the Cq value measured for the single mixed leaf sample collected at 21 dpi for the five plants included in each treatment. For the infectivity evaluation of isolate ToBRFV_G78_NRB (second experiment): plotted values for mock inoculation treatments correspond to the mean Cq value at each sampling point for the three plants included in each treatment, and plotted values for viral inoculation treatment correspond to the mean of five Cq values measured at each sampling point for the five plants included in each treatment. The standard error for each mean Cq measurement is indicated with error bars. Each experiment is plotted in a different shape: triangle for infectivity evaluation of ToBRFV_G78_RB (first experiment) and circle for infectivity evaluation of ToBRFV_G78_NRB (second experiment). Each treatment is plotted in a different color and shade combination: red color for the susceptible cultivar treatments and green color for the new resistant cultivar treatments; light shades for the mock inoculation treatments and dark shades for the ToBRFV inoculation treatments. The plot’s background colors indicate Cq range attributed to each ToBRFV titer tier.

The mock inoculation treatments of the bioassay were used as healthy controls and served as a baseline for symptom assessment. ToBRFV was not detected in any of the four mock-inoculated control treatments (in the two experiments) at any of the sampling points (**Figure 4**), and none of those mock-inoculated control plants had developed ToBRFV symptoms (**Supplementary Figures 6, 7**, **Supplementary Tables 2**, **3**). These data confirmed that no contaminations had occurred.

**Figure 5:**
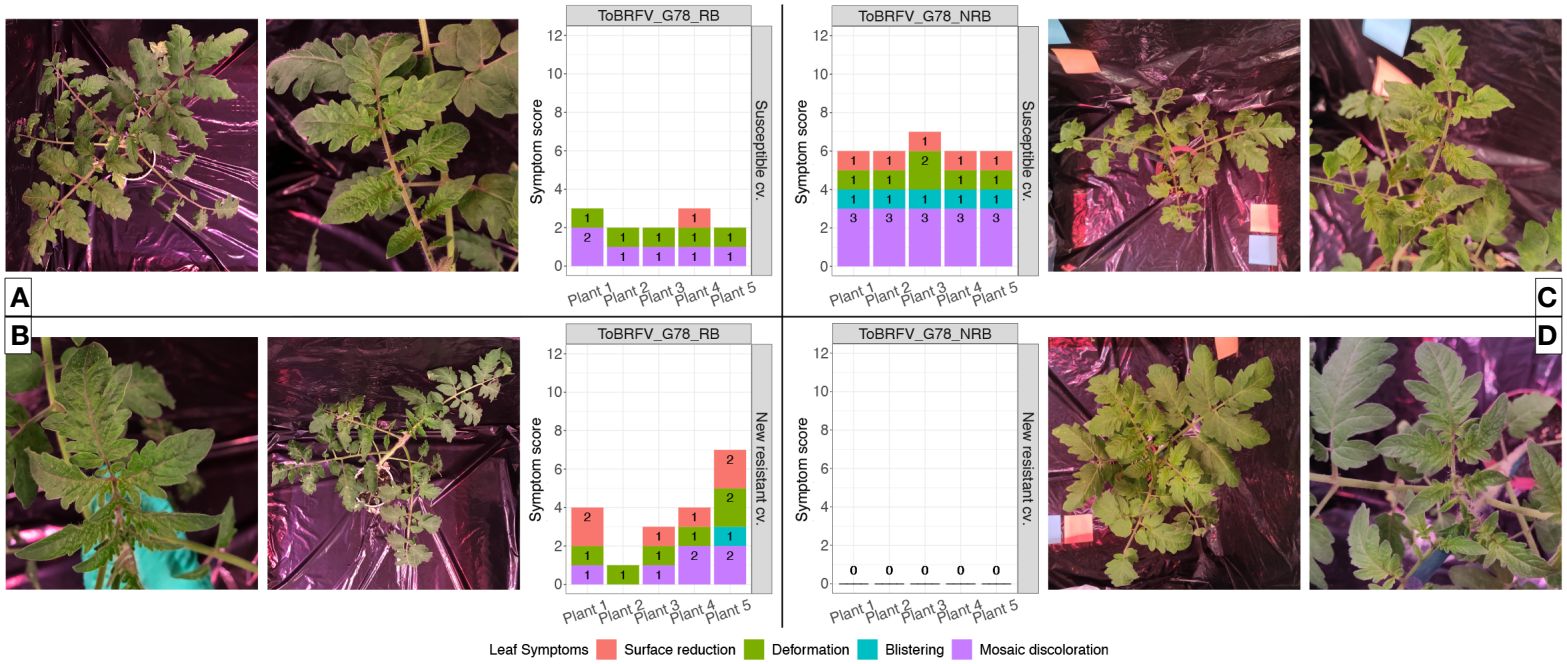
Infectivity evaluation of the newly identified isolates ToBRFV_G78_RB and ToBRFV_G78_NRB in a new resistant cultivar—leaf symptom development. **(A)** ToBRFV-susceptible plants inoculated with ToBRFV_G78_RB at 28 dpi. Left: representative pictures for leaf symptom development: plant displays moderate mosaic discoloration (light and dark green patches) as well as mild deformation (leaf tips and leaf segments start to elongate, leaf lobes become more indented, leaf shape starts to change and is no longer reminiscent of the typical tomato leaf). Right: symptom scoring per plant included in this treatment. **(B)** New resistant cv. E15A.42917 plants inoculated with ToBRFV_G78_RB at 28 dpi. Left: representative pictures for leaf symptom development: plant displays moderate mosaic discoloration (light and dark green patches) as well as mild deformation (partially segmented leaflets, pointy leaf tips) and mild leaf surface reduction (in comparison with the infection-negative controls for the experiment). Right: symptom scoring per plant included in this treatment. **(C)** ToBRFV-susceptible plants inoculated with ToBRFV_G78_NRB at 28 dpi. Left: symptom scoring per plant included in this treatment. Right: representative pictures for leaf symptom development: plant displays severe mosaic discoloration (light and dark green patches) as well as mild blistering (bubble-like formations), mild deformation (partially segmented leaflets, pointy leaf tips), and mild surface reduction (in comparison with the infection-negative controls for the experiment). **(D)** New resistant cv. E15A.42917 plants inoculated with ToBRFV_G78_NRB at 28 dpi. Left: symptom scoring per plant included in this treatment. Right: representative pictures for leaf symptom development: No ToBRFV leaf symptoms were observed on the plant. For symptom scoring bar graphs: x-axis: plants included in each treatment. y-axis: overall symptom scoring. Each leaf symptom is plotted in a different color. The score for each symptom is included in black on each bar.

The susceptible plants inoculated with both ToBRFV_G78_RB (n = 5) and ToBRFV_G78_NRB (n = 5) served as positive controls for infection. Inoculation of these susceptible plants with either isolate indeed resulted in low ToBRFV Cq values already from the first sampling point. ToBRFV Cq values remained low throughout the course of the bioassay. In the ToBRFV-susceptible plants inoculated with ToBRFV_G78_RB (n = 5), the presence of the T to G substitution at position NCBI reference genome position 5,156 was confirmed with Sanger sequencing at 28 dpi. The presence or absence of a substitution at position 5,156 did not significantly affect the viral concentration in the susceptible plants, as the Cq values of the positive controls for infection were not statistically different throughout the bioassay (p-value: 0.78) (**Figure 4**). At symptom scoring, all five susceptible plants inoculated with isolate ToBRFV_G78_RB had developed mild or moderate mosaic discoloration and displayed mild deformation. One plant also showed mild leaf surface reduction (**Figure 5A**). The leaf symptoms of susceptible plants inoculated with ToBRFV_G78_NRB at 28 dpi were more severe than those observed in the ToBRFV_G78_RB inoculated new resistant plants. All plants of the susceptible cultivar had developed severe mosaic symptoms, mild blistering, mild or moderate deformation and mild surface reduction (**Figure 5C**).

Interestingly, also the new resistant plants inoculated with ToBRFV_G78_RB displayed low Cq values for ToBRFV already at 14 dpi. These low Cq values remained stable and low until the end of the bioassay (**Figure 4**). Throughout the bioassay, the detected Cq values were significantly different from the Cq values of the mock inoculated healthy controls of both experiments (p-values: 3.55e−12, 1.67e−06), while not significantly different from the positive controls for infection of both experiments (p-values: 0.48, 0.33). At symptom scoring, four plants had developed mild or moderate mosaic discoloration, and one had mild blistering. Additionally, all five plants displayed mild or moderate deformation, and four plants showed mild or moderate leaf surface reduction (**Figure 5B**).

The new resistant plants inoculated with ToBRFV_G78_NRB did not display any ToBRFV symptoms (**Figure 5D**), and the virus was not detectable throughout the course of the bioassay (**Figure 4**). The detected Cq values were significantly different from the positive controls for infection of both experiments (p-values: 1.04e−07, 4.02e−08) and from the Cq values of the new resistant plants inoculated with ToBRFV_G78_RB (p-value: 1.87e−06). These results were in line with the observations of the screening bioassay using the ToBRFV-WT isolate.

## 4 Discussion

Recently the commercial cultivation of tomato has been critically affected by the emergence of ToBRFV, a member of the *Tobamovirus* genus breaking the dominant *Tm-2²* resistance against tobamoviruses that is present in commercial tomato cultivars. ToBRFV has a very significant impact on fruit marketability and yield (Caruso et al., 2022). In response to this threat, breeding companies have invested heavily in the rapid development of ToBRFV-resistant cultivars based on a variety of mechanisms. The first resistant cultivars are being introduced in the market since 2022 (Ykema et al., 2020, 2021; Varda et al., 2022; Gilan et al., 2023; Wanten, 2023).

In this study, the new resistant cv. E15A.42917 has been screened for resistance against a wild-type isolate of ToBRFV. This cultivar, as found in deposit accession number NCIMB 43279, harbors a new resistance against ToBRFV (Ykema et al., 2020). Cultivar resistance can be achieved with the introgression of R genes in the plant genome. The products of R genes have the ability to interact specifically with viral proteins that act as effector molecules and are encoded by viral avirulence (*avr*) genes (Dangl and Jones, 2001). The interaction can occur by direct binding of the Avr proteins to R proteins (Dangl and Jones, 2001). There are also resistance cases where the R proteins interact with the viral proteins indirectly, through recognition of viral and plant target protein complexes, which are usually part of the plant defense pathway (Dangl and Jones, 2001). The interaction between the R and Avr proteins activates the plant defense leading to suppression of infection usually via the rapid death of the infected cells causing necrotic lesions on the plant, a reaction referred to as “hypersensitive response” (Dangl and Jones, 2001; Gururani et al., 2012). The prevention of viral spread can also be achieved without the display of necrotic symptoms. In cases of “extreme resistance,” viral replication is inhibited at the infected cells (Kang et al., 2005; Spiegelman and Dinesh-Kumar, 2023). Other resistant cultivars have been described that still restrict viral spread, while allowing viral replication to some extent, which results in localized or no symptoms (Kang et al., 2005). In the current study, high Cq values for ToBRFV have been detected in new resistant cv. E15A.42917 plants inoculated with ToBRFV-WT at 14 dpi, while no viral amplification has been detected at the following sampling points (**Figure 1**). These viral detection results in combination with the absence of symptoms at 28 dpi (**Figure 2**) indicate that new resistant cv. E15A.42917 might indeed allow an initial viral amplification but restrict the viral spread. This detection could also be caused by aerosols or viral RNA residue on the plants created during the inoculation process, although young leaves were sampled.

After initial screening experiments in a growth chamber with highly controlled conditions, confirming the resistance, these new resistant cultivars have also been monitored in commercial greenhouse conditions. In general, the new resistant cultivars are planted in complete greenhouse compartments. However, in some cases, the new resistant cultivars have been planted in between susceptible cultivars. Here, such a case of interplanting is followed up in more detail (**Supplementary Figure 3**). The interplanting setup is of particular interest because in the event of an outbreak, it results in a high viral pressure on the new resistant cultivars. Plant RNA viruses, such as ToBRFV, have a high mutation rate and, in most cases, exist in infected plants as a “quasi-species,” a complex of different isolates (Harrison, 2002). The composition of the viral complex is dynamic and allows the quick selection of mutants with an increased fitness under changing host and/or environmental conditions (LaTourrette and Garcia-Ruiz, 2022). Fittingly, monitoring of the new resistant cultivars under practical conditions has shown that they perform best in commercial greenhouses where entire compartments have been only planted with them, thus efficiently separating the susceptible from the new resistant plants. In several commercial greenhouses that apply interplanting schemes, a few weeks after planting, ToBRFV symptoms have been observed both in susceptible and, unexpectedly, also in new resistant cultivars harboring the same resistance gene as the cultivar examined in this study. In the case of greenhouse 78, immediately upon symptom detection on the susceptible cultivar, leaf samples were collected from both susceptible and new resistant plants. After RT-qPCR analysis, both sample types had low Cq values for ToBRFV (**Table 1**) suggesting that the resistance offered by the new resistant cultivar was likely broken, and thus, it could not prevent infection in the greenhouse setting.

The durability and specificity of the resistance offered by R genes is determined by their products’ ability to recognize viral effectors and is usually limited to closely related viruses expressing similar Avr proteins (Gururani et al., 2012; Märkle et al., 2022). Therefore, nucleotide substitutions, and especially non-synonymous substitutions resulting in amino acid changes in the *avr* genes, can strongly impact the efficacy of an R gene (Moreno-Pérez et al., 2016). The resistance offered by some R genes, like the tomato gene *Sw-5*, the potato genes *Nb* and *Nx*, and the melon gene *Cvy-1^1^*, has been found to be broken by a single amino acid change in the cell-to-cell movement protein (NSM) of tomato spotted wilt virus (TSWV) (Batuman et al., 2017), the movement protein (MP) of potato virus X (PVX) (Harrison, 2002), the coat protein (CP) of PVX (Harrison, 2002), and the viral protein genome-linked (VPg) of cucumber vein yellowing virus (CVYV) (Desbiez et al., 2022a), respectively. Resistance breaking for R genes, like potato gene *Rx1* and sugar beet gene *Rz1*, on the other hand, requires the accumulation of multiple amino acid changes in the recognized Avr proteins (Harrison, 2002; Liebe et al., 2023).

Efforts to decipher the mechanism behind *Tm-2^2^* resistance have led to the identification of the tobamovirus MP as a viral effector and have connected changes in its amino acid sequence with resistance breaking (Weber and Pfitzner, 1998). Following the emergence of ToBRFV, genomic analysis has revealed 12 nucleotide substitutions in the MP-coding part of its genome that are likely associated with breaking of the *Tm-2^2^* resistance (Maayan et al., 2018). Additionally, transient expression of the ToBRFV MP leads to overcoming of the *Tm-2^2^* resistance (Hak and Spiegelman, 2021). Breaking of the *Tm-2^2^* resistance has also been achieved in inoculation assays with a recombinant ToMV virus, where the original MP is replaced with the ToBRFV MP (Hak and Spiegelman, 2021).

Considering these data, one leaf sample each from the susceptible and the new resistant cultivar have been selected for HTS analysis, and the ToBRFV sequences obtained have been named ToBRFV_G78_NRB and ToBRFV_G78_RB, respectively. The isolates have been inspected for nucleotide substitutions that could potentially result in resistance breaking in the new resistant cultivar. The sequence comparison shows a single nucleotide difference at position 5,144 of the two genomes, aligning with position 5,156 of the NCBI reference sequence for ToBRFV. The ToBRFV_G78_NRB sequence has the wild-type base (T) on that position, while the ToBRFV_G78_RB had a substitution with base G (**Figure 3**). This nucleotide substitution leads to the amino acid change N82K in the MP of the isolate of the new resistant cultivar (**Figure 3**). In a recent study, *S. habrochaites* wild solanaceous genotypes that showed resistance were subjected to repeated inoculation rounds with ToBRFV (Jewehan et al., 2022). During this test, a ToBRFV mutant was identified (isolate ToBRFV-Tom2M-Jo) that was also able to break the newly identified resistance (Jewehan et al., 2022). Molecular characterization of the isolate associated with the resistance breaking showed two amino acid changes at positions 22 and 82 of the MP (Jewehan et al., 2022). Interestingly, the cultivars examined in our study harbor a resistance against ToBRFV that was introgressed from the same wild solanaceous species, *S. habrochaites* (Ykema et al., 2020). Combining the data from Jewehan’s study with the data obtained in the present study confirm that residue 82 in the MP of ToBRFV is crucial for the development of resistance and is, on its own, sufficient to confer the resistant-breaking phenotype. Additionally, the combined examination of the findings indicates that the ToBRFV resistance observed in both cases is possibly originating from the same or allelic genes. A subsequent comparison of the ToBRFV_G78_RB nucleotide sequence with all the publicly available ToBRFV full genome sequences shows that the nucleotide substitution that leads to the amino acid change N82K in the MP of the virus was identified only for ToBRFV_G78_RB and ToBRFV-Tom2M-Jo (**Supplementary Figure 5**). This comparison also reveals that the nucleotide substitution that leads to the amino acid change in position 22 of the MP, which was identified in Jehewan’s study, is unique to ToBRFV-Tom2M-Jo. In the absence of an isolate with an amino acid change in residue 22 of the MP alone, the role this residue plays in the development of the resistance-breaking phenotype remains to be experimentally investigated.

A second screening assay has been set up with ToBRFV_G78_RB using ToBRFV_G78_NRB as a control to confirm that the single amino acid change N82K in the MP can break the resistance of the new resistant cultivar. The new resistant plants inoculated with ToBRFV_G78_NRB, similarly with the new resistant plants inoculated with ToBRFV-WT in the first screening assay, show no ToBRFV detection throughout the assay and no viral symptoms (**Figures 4**, **5**). On the contrary, the new resistant plants inoculated with ToBRFV_G78_RB are symptomatic, and low Cq values are measured that match the Cq values observed in the susceptible plants that served as positive controls (**Figures 4**, **5**). It has also been observed that, although the Cq values of ToBRFV_G78_NRB and ToBRFV_G78_RB are comparable in the respectively inoculated susceptible plants, the ToBRFV_G78_NRB-induced symptoms are more severe than those caused by ToBRFV_G78_RB (**Figures 4**, **5**). These results suggest that the amino acid change N82K in the MP that allows the isolate to overcome the resistance and could potentially have an effect on the symptom severity in this cultivar. Single amino acid changes have been observed to reduce the infectivity and pathogenicity of plant viruses, such as tomato torrado virus (Wieczorek and Obrepalska-Steplowska, 2016) and zucchini yellow mosaic virus (Desbiez et al., 2022b).

The durability of resistant cultivars against plant viruses can be affected by many factors such as the type and number of resistance genes, the mutation rate of the targeted viruses, the selection pressure applied to the virus by the environment, and the virus fitness cost of different mutations (Harrison, 2002; LaTourrette and Garcia-Ruiz, 2022). Although it is important to consider all those parameters during the development of a new resistant cultivar and the initial confirmatory assays, the thorough assessment of the cultivar’s performance in practical conditions is valuable and can reveal new challenges in establishing an efficient plant virus management method. In this study, the initial screening of the new resistant cultivars has confirmed the new resistance against ToBRFV under controlled conditions. However, their use in standard tomato production conditions has revealed the importance of maintaining viral pressure to a minimum by combining the implementation of hygiene measures, vigilant monitoring, and careful crop management, among others. Interplanting the resistant cultivars among susceptible plants increased the surrounding viral pressure and facilitated the emergence of a resistance-breaking isolate. Resistant cultivars are a major step forward in the effort to protect tomato cultivation against ToBRFV, but it remains important to include them into an integrated management approach targeted at keeping viral pressure to a minimum.

## Data availability statement

The datasets presented in this study can be found in online repositories. The names of the repository/repositories and accession number(s) can be found in the article/**Supplementary Material**.

## Author contributions

ZZ: Conceptualization, Data curation, Formal analysis, Funding acquisition, Investigation, Software, Visualization, Writing – original draft, Writing – review & editing. LG: Conceptualization, Funding acquisition, Investigation, Writing – review & editing. EV: Conceptualization, Funding acquisition, Investigation, Methodology, Writing – review & editing. CV: Conceptualization, Funding acquisition, Methodology, Project administration, Resources, Supervision, Writing – review & editing. JM: Conceptualization, Funding acquisition, Methodology, Project administration, Resources, Supervision, Writing – review & editing.
