# Supplementary material for: Single amino acid change in tomato brown rugose fruit virus breaks virus-specific resistance in new resistant tomato cultivar
Source: Front Plant Sci. 2024 May 7;15:1382862. doi: 10.3389/fpls.2024.1382862 (PMC11106371; doi:10.3389/fpls.2024.1382862)
Supplement: Supplementary file 1 [file DataSheet_1.docx]

Supplementary Material

1. **Supplementary Figures and Tables**
   1. **Supplementary Figures**

**
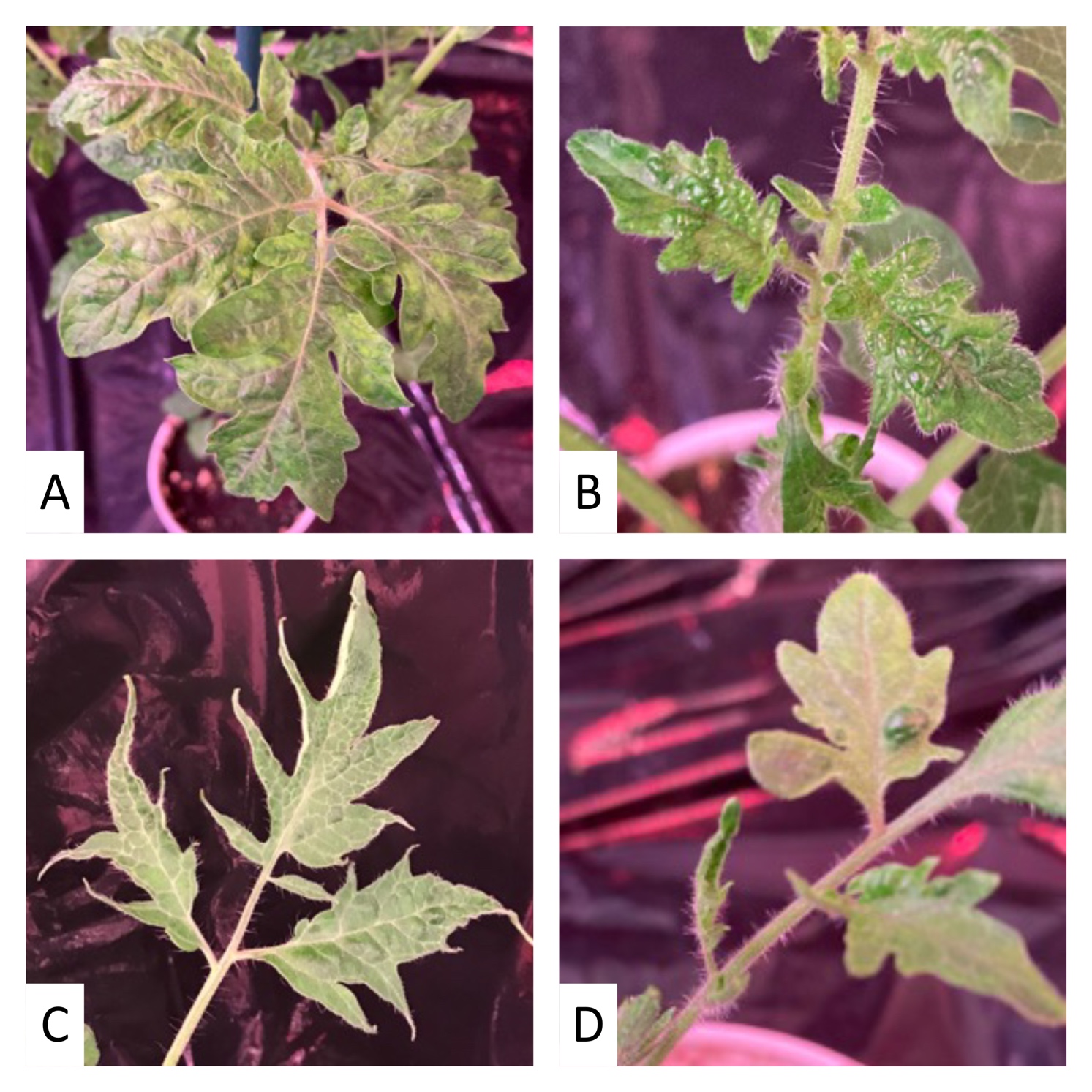
**

**Supplementary Figure 1.** ToBRFV symptom scoring scale – Symptom presence. The scale assesses the development of the following symptoms on the leaves of tomato plants: (A) mosaic discoloration – presence of light and dark green patches on the leaves, (B) blistering – presence of bubble-like formations on the leaves, (C) deformation – leaf shape not typical for tomato plants (e.g. presence of pointy leaf ends, asymmetrical leaves, elongated and narrow leaves), (D) surface reduction – leaf size reduced in comparison with negative control leaf size.

**
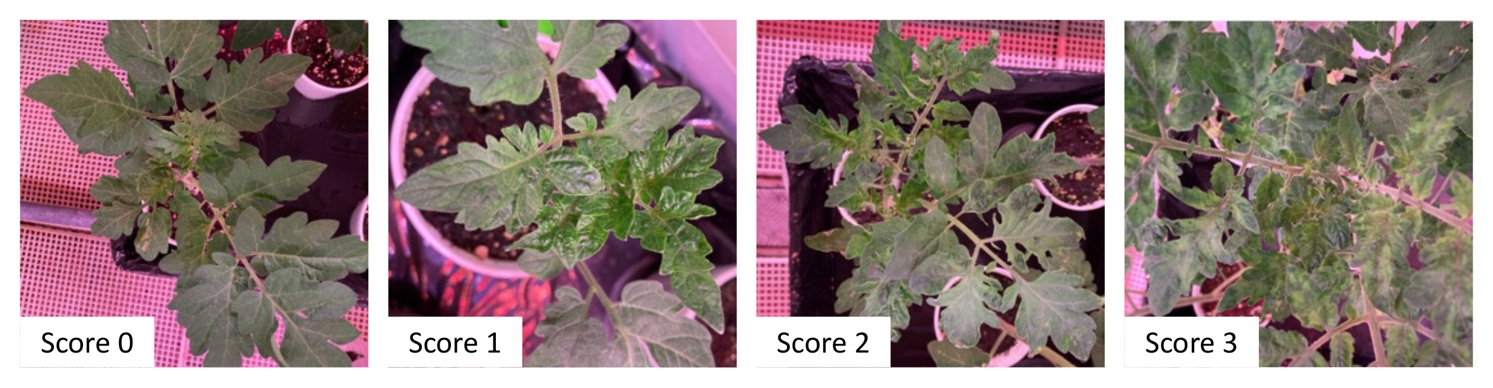
**

**Supplementary Figure 2.** ToBRFV symptom scoring scale – Symptom severity. The scale assesses the severity of each of the developed symptoms on the leaves of tomato plants.The figure presents an example of the scoring range for the mosaic discoloration symptom. Score 0: Absence of light or dark green patches on the leaves. Score 1: localized presence of light and dark green patches on the leaf – the discoloration has mild intensity and/or is present in a few leaves. Score 2: light and dark green patches cover large areas of the leaf – the discoloration has moderate intesity and/or is present in most leaves. Score 3: light and dark patches cover the entirety of the leaf – the discoloration has high intensity and/or is present in all the leaves.

**
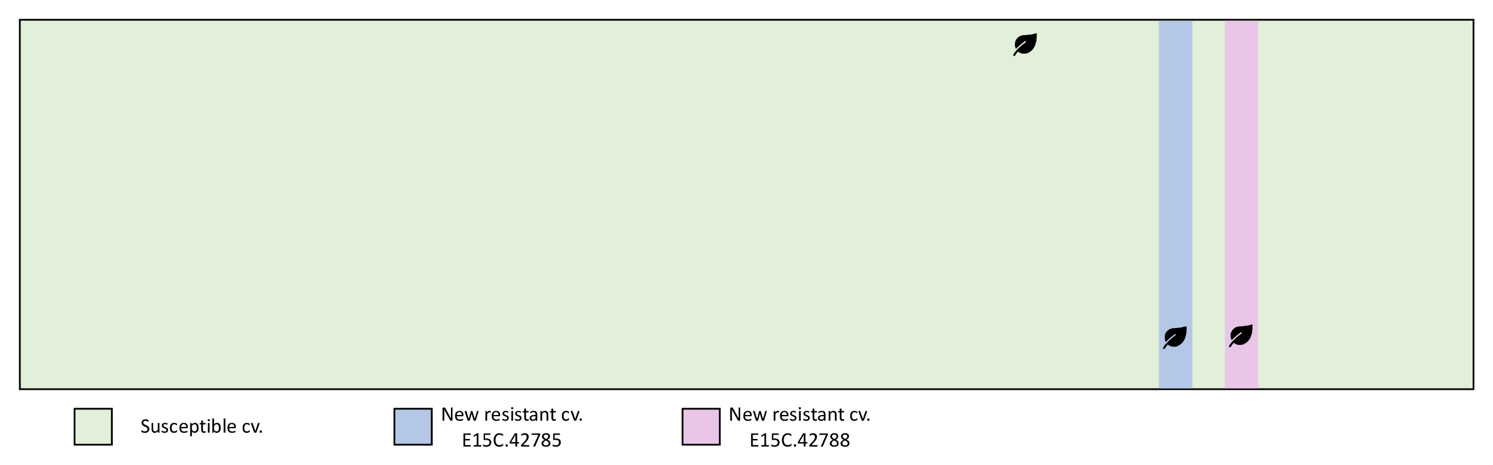
**

**Supplementary Figure 3.** Commercial greenhouse 78 planting scheme overview. The planting arrangement is shown in color: Green for the susceptible cv. Sunstream, blue for the new resistant cv. E15C.42785, and pink for the new resistant cv. E15C.42788. The sampling positions are shown by a leaf icon.

**
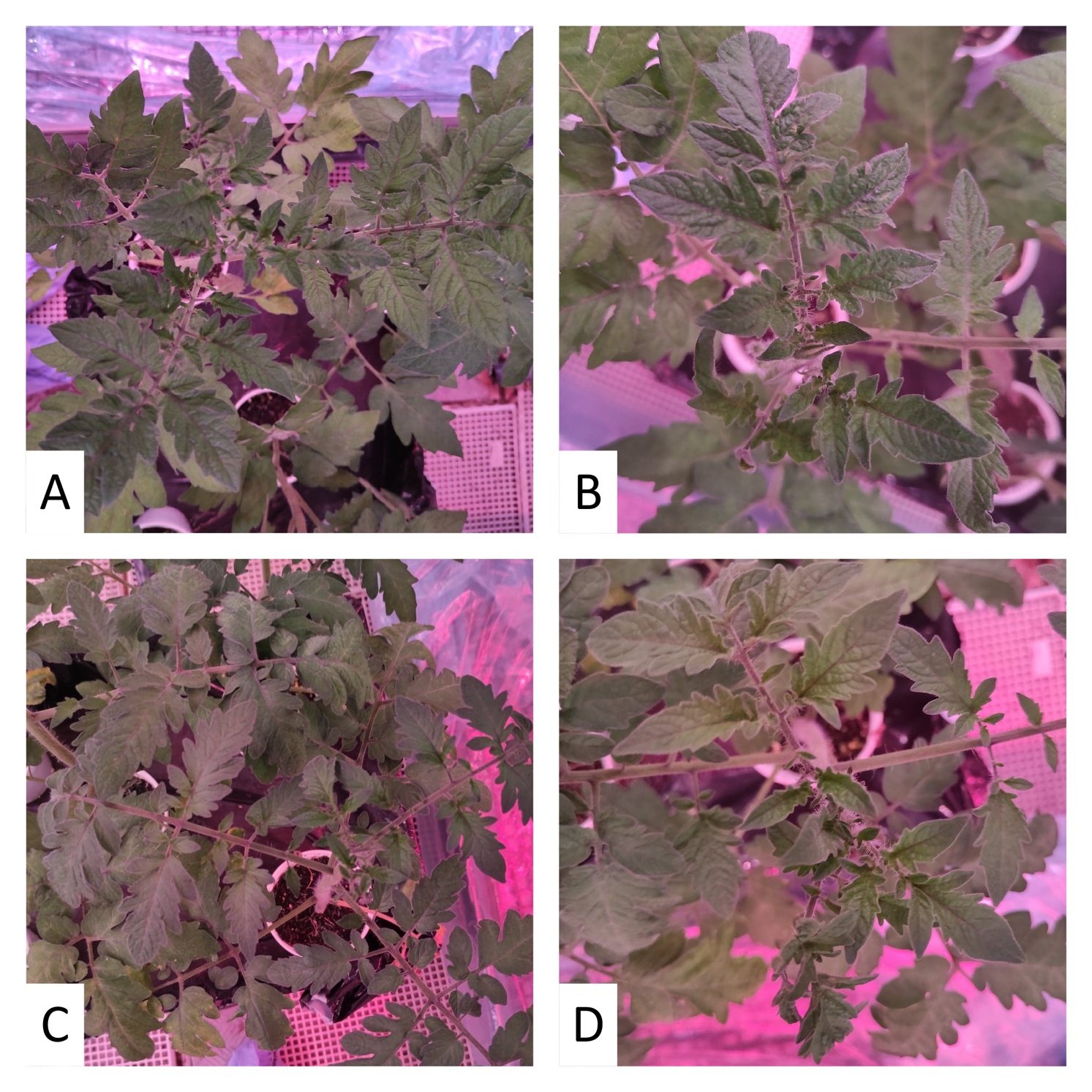
**

**Supplementary Figure 4.** New resistant cultivar screening against ToBRFV-WT – Infection negative controls. (A), (B) ToBRFV susceptible plants mock inoculated, at 28dpi, no ToBRFV symptoms developed. (C), (D) New resistant cv. E15A.42917 plants mock inoculated, at 28dpi no ToBRFV symptoms developed.

**
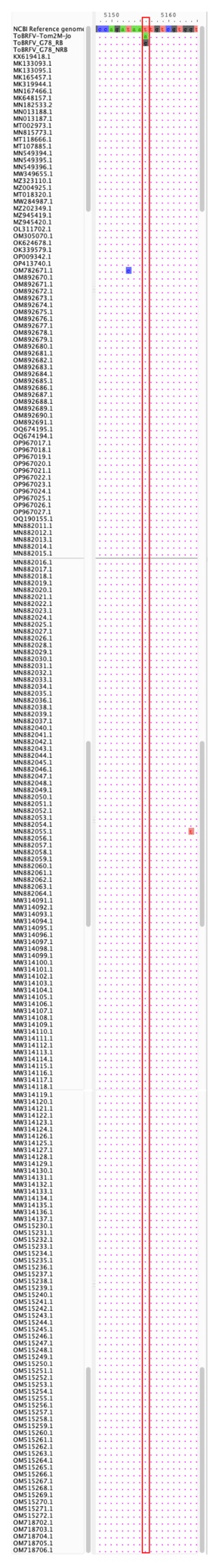
**

**Supplementary Figure 5.** Comparison of the publicly available ToBRFV full genome sequences in NCBI GenBank (data downloaded on 30/06/23) at reference position 5156. Nucleotides that are identical to the reference genome are represented with a pink dot. Substitutions are represented with the alternative nucleotide. Position 5156 is highlighted with a red rectangle.

**
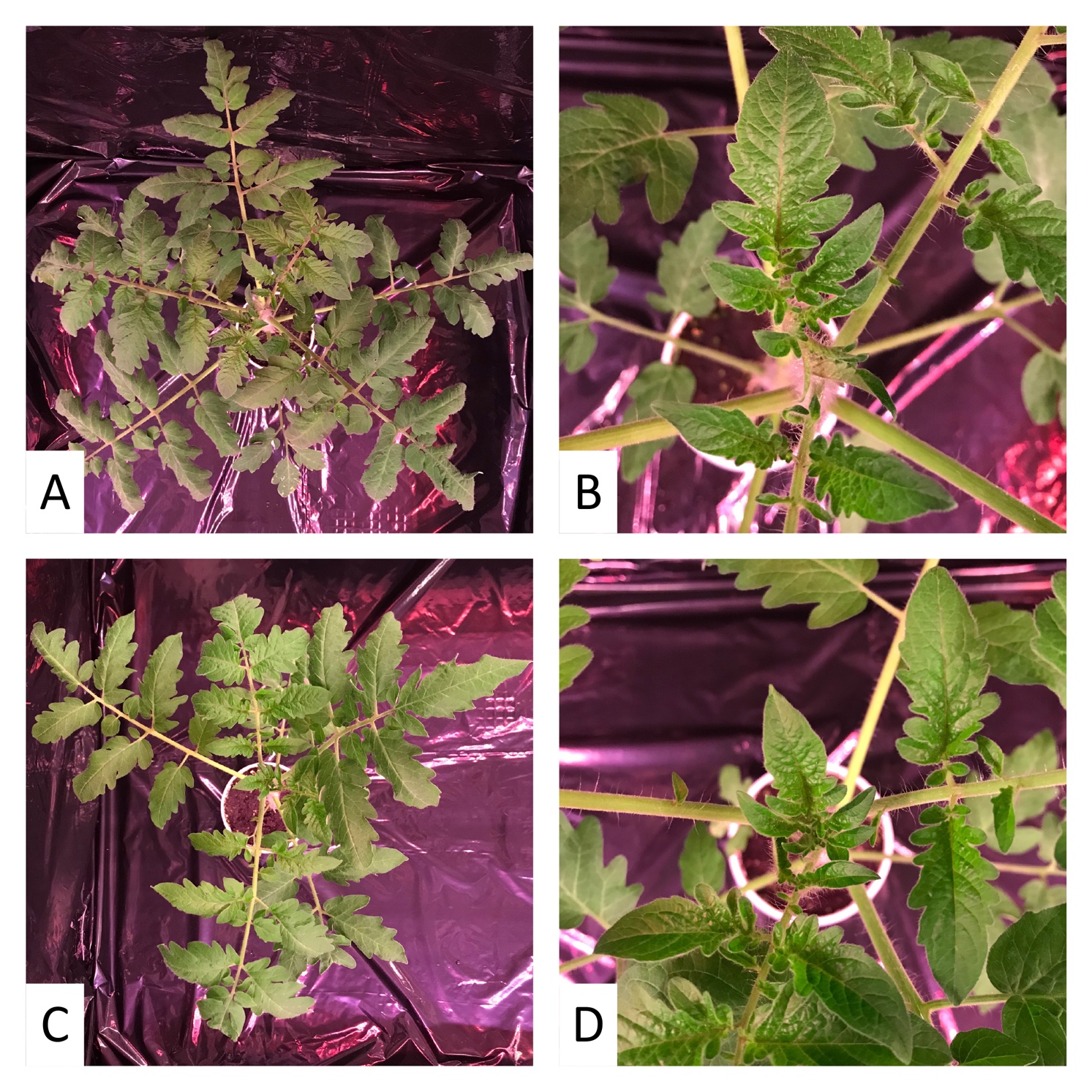
**

**Supplementary Figure 6.** Infectivity evaluation of the newly identified isolates ToBRFV_G78_RB and ToBRFV_G78_NRB in a new resistant cultivar – First experiment – Infection negative controls. (A), (B) ToBRFV susceptible plants mock inoculated, at 28dpi, no ToBRFV symptoms developed. (C), (D) New resistant cv. E15A.42917 plants mock inoculated, at 28dpi, no ToBRFV symptoms developed.

**
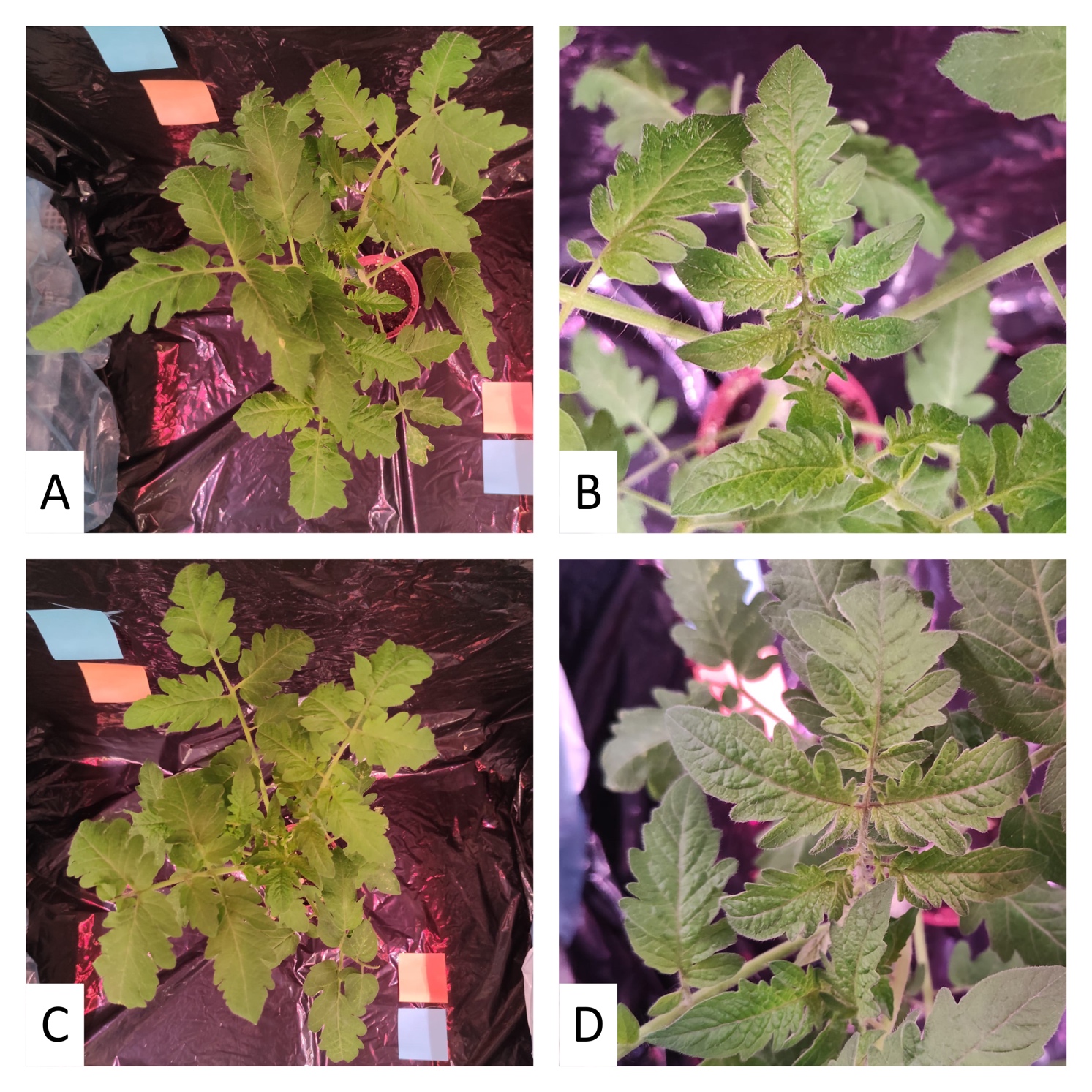
**

**Supplementary Figure 7.** Infectivity evaluation of the newly identified isolates ToBRFV_G78_RB and ToBRFV_G78_NRB in a new resistant cultivar – Second experiment – Infection negative controls. (A), (B) ToBRFV susceptible plants mock inoculated, at 28dpi, no ToBRFV symptoms developed. (C), (D) New resistant cv. E15A.42917 plants mock inoculated, at 28dpi, no ToBRFV symptoms developed.

- 1. **Supplementary Tables**

**Supplementary Table 1.** New resistant cultivar screening against ToBRFV-WT – Leaf symptom scoring at 28dpi.

| **Treatment** | **Plant** | **Individual symptom scoring** | | | | **Overall score** |
| --- | --- | --- | --- | --- | --- | --- |
|  |  | **Mosaic discoloration** | **Blistering** | **Deformation** | **Surface reduction** |  |
| **A: Susceptible cultivar - Mock inoculation** | **1** | 0 | 0 | 0 | 0 | **0** |
|  | **2** | 0 | 0 | 0 | 0 | **0** |
|  | **3** | 0 | 0 | 0 | 0 | **0** |
|  | **4** | 0 | 0 | 0 | 0 | **0** |
|  | **5** | 0 | 0 | 0 | 0 | **0** |
| **B: Susceptible cultivar - ToBRFV-WT** | **1** | 3 | 1 | 1 | 1 | **6** |
|  | **2** | 3 | 0 | 1 | 1 | **5** |
|  | **3** | 3 | 1 | 2 | 1 | **7** |
|  | **4** | 3 | 0 | 1 | 1 | **5** |
|  | **5** | 3 | 0 | 1 | 1 | **5** |
| **C: New resistant cultivar - Mock inoculation** | **1** | 0 | 0 | 0 | 0 | **0** |
|  | **2** | 0 | 0 | 0 | 0 | **0** |
|  | **3** | 0 | 0 | 0 | 0 | **0** |
|  | **4** | 0 | 0 | 0 | 0 | **0** |
|  | **5** | 0 | 0 | 0 | 0 | **0** |
| **D: New resistant cultivar - ToBRFV-WT** | **1** | 0 | 0 | 0 | 0 | **0** |
|  | **2** | 0 | 0 | 0 | 0 | **0** |
|  | **3** | 0 | 0 | 0 | 0 | **0** |
|  | **4** | 0 | 0 | 0 | 0 | **0** |
|  | **5** | 0 | 0 | 0 | 0 | **0** |

**Supplementary Table 2.** Infectivity evaluation of the newly identified isolates ToBRFV_G78_RB and ToBRFV_G78_NRB in a new resistant cultivar – First experiment – Leaf symptom scoring at 28dpi.

| **Treatment** | **Plant** | **Individual symptom scoring** | | | | **Overall score** |
| --- | --- | --- | --- | --- | --- | --- |
|  |  | **Mosaic discoloration** | **Blistering** | **Deformation** | **Surface reduction** |  |
| **A: Susceptible cultivar - Mock inoculation** | **1** | 0 | 0 | 0 | 0 | **0** |
|  | **2** | 0 | 0 | 0 | 0 | **0** |
|  | **3** | 0 | 0 | 0 | 0 | **0** |
|  | **4** | 0 | 0 | 0 | 0 | **0** |
|  | **5** | 0 | 0 | 0 | 0 | **0** |
| **B: Susceptible cultivar - ToBRFV_G78_RB** | **1** | 2 | 0 | 1 | 0 | **3** |
|  | **2** | 1 | 0 | 1 | 0 | **2** |
|  | **3** | 1 | 0 | 1 | 0 | **2** |
|  | **4** | 1 | 0 | 1 | 1 | **3** |
|  | **5** | 1 | 0 | 1 | 0 | **2** |
| **C: New resistant cultivar - Mock inoculation** | **1** | 0 | 0 | 0 | 0 | **0** |
|  | **2** | 0 | 0 | 0 | 0 | **0** |
|  | **3** | 0 | 0 | 0 | 0 | **0** |
|  | **4** | 0 | 0 | 0 | 0 | **0** |
|  | **5** | 0 | 0 | 0 | 0 | **0** |
| **D: New resistant cultivar - ToBRFV_G78_RB** | **1** | 1 | 0 | 1 | 2 | **4** |
|  | **2** | 0 | 0 | 1 | 0 | **1** |
|  | **3** | 1 | 0 | 1 | 1 | **3** |
|  | **4** | 2 | 0 | 1 | 1 | **4** |
|  | **5** | 2 | 1 | 2 | 2 | **7** |

**Supplementary Table 3.** Infectivity evaluation of the newly identified isolates ToBRFV_G78_RB and ToBRFV_G78_NRB in a new resistant cultivar – Second experiment – Leaf symptom scoring at 28dpi.

| **Treatment** | **Plant** | **Individual symptom scoring** | | | | **Overall score** |
| --- | --- | --- | --- | --- | --- | --- |
|  |  | **Mosaic discoloration** | **Blistering** | **Deformation** | **Surface reduction** |  |
| **E: Susceptible cultivar - Mock inoculation** | **1** | 0 | 0 | 0 | 0 | **0** |
|  | **2** | 0 | 0 | 0 | 0 | **0** |
|  | **3** | 0 | 0 | 0 | 0 | **0** |
| **F: Susceptible cultivar - ToBRFV_G78_NRB** | **1** | 3 | 1 | 1 | 1 | **6** |
|  | **2** | 3 | 1 | 1 | 1 | **6** |
|  | **3** | 3 | 1 | 2 | 1 | **7** |
|  | **4** | 3 | 1 | 1 | 1 | **6** |
|  | **5** | 3 | 1 | 1 | 1 | **6** |
| **G: New resistant cultivar - Mock inoculation** | **1** | 0 | 0 | 0 | 0 | **0** |
|  | **2** | 0 | 0 | 0 | 0 | **0** |
|  | **3** | 0 | 0 | 0 | 0 | **0** |
| **H: New resistant cultivar - ToBRFV_G78_NRB** | **1** | 0 | 0 | 0 | 0 | **0** |
|  | **2** | 0 | 0 | 0 | 0 | **0** |
|  | **3** | 0 | 0 | 0 | 0 | **0** |
|  | **4** | 0 | 0 | 0 | 0 | **0** |
|  | **5** | 0 | 0 | 0 | 0 | **0** |
